# Role of HCA_2_ in Regulating Intestinal Homeostasis and Suppressing Colon Carcinogenesis

**DOI:** 10.3389/fimmu.2021.606384

**Published:** 2021-02-23

**Authors:** Zhuoyue Li, Kayleen J. McCafferty, Robert L. Judd

**Affiliations:** Department of Anatomy, Physiology and Pharmacology, College of Veterinary Medicine, Auburn University, Auburn, AL, United States

**Keywords:** HCA_2_, intestinal homeostasis, anti-inflammatory, intestinal inflammation, colon cancer, mucosal immunity, microbiota

## Abstract

Hydroxycarboxylic acid receptor 2 (HCA_2_) is vital for sensing intermediates of metabolism, including β-hydroxybutyrate and butyrate. It also regulates profound anti-inflammatory effects in various tissues, indicating that HCA_2_ may serve as an essential therapeutic target for mediating inflammation-associated diseases. Butyrate and niacin, endogenous and exogenous ligands of HCA_2_, have been reported to play an essential role in maintaining intestinal homeostasis. HCA_2_, predominantly expressed in diverse immune cells, is also present in intestinal epithelial cells (IECs), where it regulates the intricate communication network between diet, microbiota, and immune cells. This review summarizes the physiological role of HCA_2_ in intestinal homeostasis and its pathological role in intestinal inflammation and cancer.

## Introduction

The intestinal tract is an organ system with specialized architecture that functions to digest food, and extract and absorb energy and nutrients. It also secretes over 20 different hormones and harbors more than 640 different species of bacteria (1). Physiological and pathophysiological events that trigger the breakdown of intestinal homeostasis negatively impact intestinal health, and may result in intestinal disorders including inflammatory bowel disease (IBD) and colitis-associated cancer. IBD is a chronic and life-threating disease characterized by prolonged inflammation of the digestive tract (2, 3). IBD encompasses two conditions, Crohn's disease and ulcerative colitis. Crohn's disease can affect any part and layer of the gastrointestinal tract, while ulcerative colitis is usually limited to the innermost layer of the colon and rectum (4). Both Crohn's disease and ulcerative colitis are characterized by episodes of fatigue, abdominal cramping, rectal bleeding, diarrhea, weight loss, and the influx of immune cells that produce cytokines, proteolytic enzymes, and free radicals (5, 6). Patients with IBD are at increased risk of developing colitis-associated cancer which is difficult to treat and has high mortality (>50%) (7, 8). In 2015, an estimated 1.3% of US adults reported living with IBD, with cases increasing worldwide (9, 10). The global spread of IBD is associated with the host genetic background, intestinal microbiota, diets, environments and immunological dysregulation (4, 11, 12).

The intestinal tract represents the largest compartment of the immune system in the body (13), with intestinal health implicated in controlling disease development not only within itself but also throughout the body. To maintain intestinal homeostasis, a multi-pronged approach including the immune system, microbial ecosystem and diet is necessary. A versatile receptor, hydroxycarboxylic acid receptor 2 (HCA_2_), is capable of both nutrient sensing and immunomodulation, lending to its popularity as a potential target for the promotion of intestine health.

In 1993, HCA_2_ was identified as an orphan receptor (GPR109A) (14, 15), and later described in mice as a “protein upregulated in macrophages by interferon-gamma (IFN-γ)” (PUMA-G) (16). In 2003, several studies reported that HCA_2_ is a receptor for niacin and functions to mediate its antilipolytic effects in adipocytes (17–19). Benyó et al. and Hanson et al. subsequently demonstrated that binding of niacin to HCA_2_ on Langerhans cells and keratinocytes is also responsible for the niacin-induced cutaneous flushing reaction, involving release of prostaglandin D_2_ (PGD_2_) and prostaglandin E_2_ (PGE_2_) (20, 21). In 2005, the ketone body β-hydroxybutyrate (β-OHB) was identified as an endogenous ligand of HCA_2_ (22). This resulted in the deorphanization of the receptor, which was subsequently renamed hydroxycarboxylic acid receptor 2 (HCA_2_) (23). Most recently, butyrate, a short-chain fatty acid (SCFA) bacterial product in the colon lumen generated at high concentrations (10–20 mM) from dietary fiber fermentation, was recognized as an endogenous ligand of HCA_2_ (24). Butyrate activation of HCA_2_ plays an important role in the maintenance of intestinal homeostasis (24). New synthetic ligands of HCA_2_ have been developed, such as acipimox, GSK256073 and derivatives of pyrazole-3-carboxylic acid or cyclopentapyrazole (25–27).

HCA_2_ is widely expressed in various tissues and cell types, including adipose tissue, spleen, lung, lymph node and intestine. HCA_2_ is predominantly expressed not only in both white and brown adipocytes, but also in diverse immune cells, including dendritic cells (DCs), monocytes, macrophages, neutrophils and epidermal Langerhans cells, but not lymphocytes (16, 18, 21, 28, 29). Interestingly, several cytokines show the ability to regulate the expression of HCA_2_ in immune cells. HCA_2_ expression is upregulated in macrophages and monocytes after IFN-γ treatment (16), and the expression of HCA_2_ in macrophages is significantly increased by proinflammatory stimulants lipopolysaccharide (LPS), interleukin (IL)-6 and IL-1β (30). Colony-stimulating factor 2 (CSF2) increases HCA_2_ expression level in neutrophils (29). HCA_2_ is also present in intestinal epithelial cells (IECs), retinal pigment epithelium, hepatocytes, keratinocytes and microglia (21, 31–34). Notably, both mRNA and protein levels of HCA_2_ in IECs are drastically reduced in germ-free mice compared to conventional mice, due to the absence of gut bacteria. These changes are reversed when the intestinal tract of germ-free mice is re-colonized (35).

**Figure 1:**
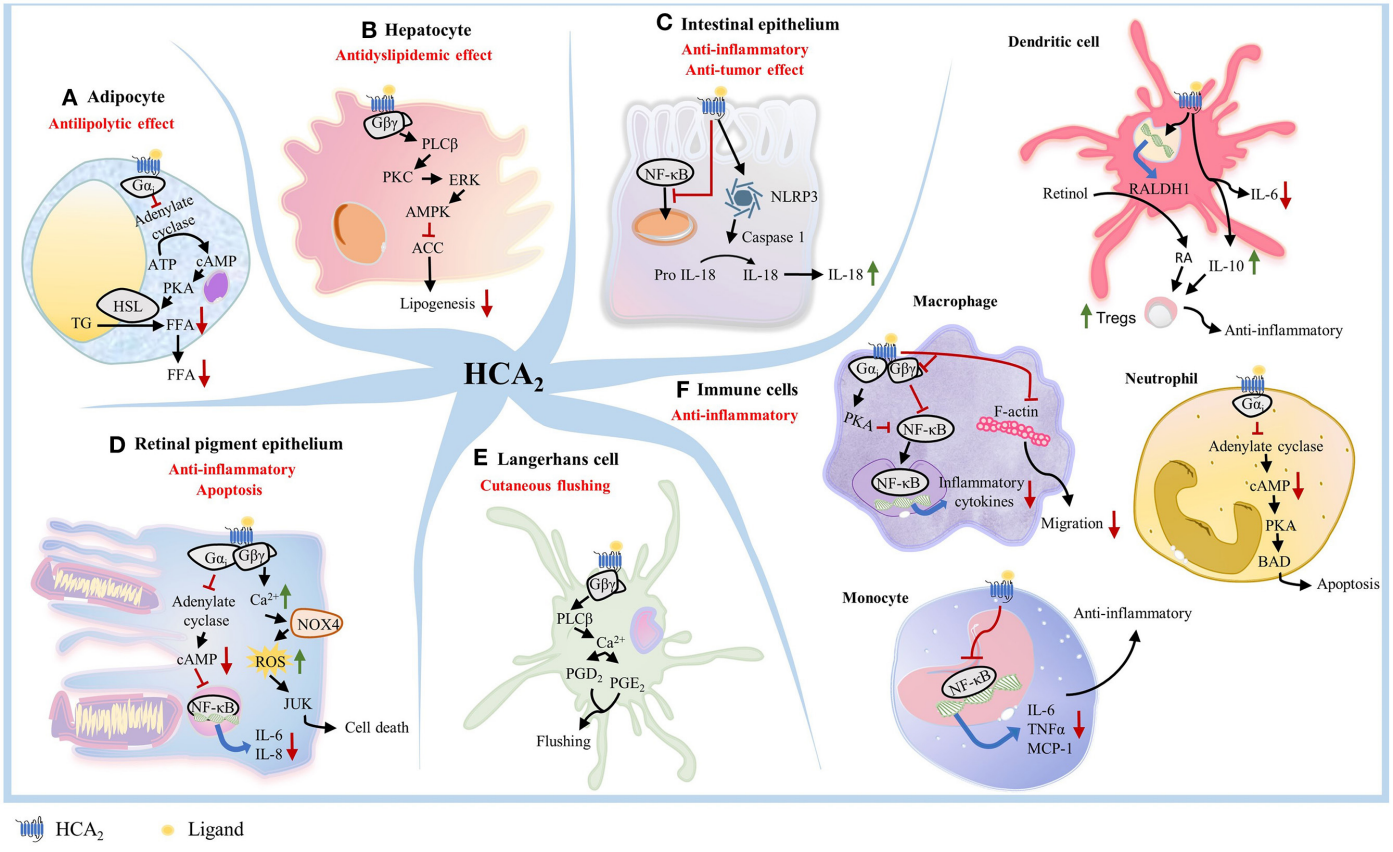
HCA_2_ triggers different downstream signaling pathway in different cell types. **(A)** In adipocytes, activation of HCA_2_ triggers a Gαi-mediated inhibition of adenylate cyclase activity, which leads to lower intracellular cAMP levels, reduced protein kinase A (PKA) activity, and further reduces the activity of hormone sensitive lipase (HSL), an important lipolytic enzyme. This inhibition of lipolysis results in a decreased release of free fatty acids into the circulation. **(B)** In hepatocytes, activation of HCA_2_ mediates the protein kinase C (PKC)-extracellular signal-regulated kinase (ERK) signaling pathway, leading to phosphorylation of AMP-activated protein kinase (AMPK) and inhibition of acetyl CoA carboxylase (ACC). This results in an inhibition of hepatic *de novo* lipogenesis and a remarkable decrease of lipid accumulation in liver. **(C)** In colonocytes, ligand binding to HCA_2_ suppresses NF-κB activation and activates NLRP3 inflammasome, which recruits caspase-1 and promotes the maturation of IL-18 for secretion. **(D)** In retinal pigment epithelium, HCA_2_ exerts either an anti-inflammatory response through the Gαi/cAMP/NF-κB pathway, or apoptosis through the Gβγ/Ca^2+^/NOX4/ROS/JNK pathway. **(E)** Within Langerhans cells, HCA_2_-mediated Gα_i_ activation primarily results in the Gβγ-complex released from activated Gα_i_, thereby increasing intracellular calcium concentration by mobilizing Ca^2+^ release from the endoplasmic reticulum and ultimately driving the formation of PGD_2_ and PGE_2_, which are released to the dermal layer and cause cutaneous flushing reaction. **(F)** In macrophages, activation of HCA_2_ involves inhibition of NF-κB, thereby exerting an anti-inflammatory effec. HCA_2_ activation in macrophages also represses F-actin and blocks Gβγ signaling to inhibit chemokine-induced migration of macrophages. HCA_2_ also shows anti-inflammatory effects in Parkinson's disease models by inhibiting the phosphorylation of the NF-κB p65 signaling pathway in microglia. HCA_2_ suppresses LPS-induced NF-κB activation in human monocytes, resulting in decreased transcription of IL-6, TNFα, and MCP-1. In DCs, HCA_2_ activation decreases IL-6 level and increases IL-10 level, also upregulates expression of RALDH1, which synthesizes RA from retinol. RA and IL-10 promote Treg cell proliferation. In neutrophils, niacin-mediated HCA_2_ activation inhibits PKA activity and subsequently increase BAD levels, which drives apoptosis of neutrophils.

While the anti-lipolytic effects of HCA_2_ are well-known, more recent studies have demonstrated that activation of HCA_2_ by endogenous and exogenous ligands is associated with anti-inflammatory effects in numerous disease states (25, 31, 36–41). Early studies showed that activation of HCA_2_ in various cell types could trigger different downstream signaling events and effects (26) (Figures 1A–F). In adipocytes, activation of HCA_2_ inhibits lipolysis (18, 42, 43) (Figure 1A). In hepatocytes, HCA_2_ mediates hepatic *de novo* lipogenesis and decreases lipid accumulation in liver (44, 45) (Figure 1B). In IECs, ligand binding to HCA_2_ activates NOD-, LRR- and pyrin domain-containing protein 3 (NLRP3) inflammasome, which promotes the maturation of IL-18 for secretion (46, 47) (Figure 1C). IL-18 is a critical effector molecule in intestinal disorders and is required for IEC proliferation (48). HCA_2_ also suppresses basal and LPS-induced nuclear factor-kappa B (NF-κB) activation in normal and cancer colonocytes (24) (Figure 1C). In retinal pigment epithelium, HCA_2_ exerts dual effects depending on the concentration of the agonist. 4-hydroxynonenal, an HCA_2_ agonist, can induce either an anti-inflammatory response or apoptosis (49) (Figure 1D). In Langerhans cells, HCA_2_ causes cutaneous flushing reaction (20, 21) (Figure 1E). In macrophages, activation of HCA_2_ exerts an anti-inflammatory effect (50, 51) (Figure 1F). HCA_2_ also represses chemokine-induced migration of macrophages (30) (Figure 1F). HCA_2_ shows anti-inflammatory effects in microglia and human monocytes (52–54) (Figure 1F). In DCs, HCA_2_ activation decreases IL-6 levels and increases IL-10 levels and upregulates expression of RALDH1, which synthesizes retinoic acid (RA) from retinol. RA is necessary for promoting regulatory T cells (Tregs) function and proliferation, especially in the gut in both murine and human DCs (55–57) (Figure 1F). In neutrophils, niacin-mediated HCA_2_ activation increases Bcl-2 associated agonist of cell death (BAD) levels, a pro-apoptotic member of the Bcl-2 family (58) (Figure 1F). Collectively, these studies clearly demonstrate that HCA_2_ plays a critical role in nutrient sensing and host protection against pro-inflammatory insults in multiple cell types using various signaling mechanisms.

## Intestinal Homeostasis and HCA_2_: Immune Cells, Intestinal Epithelium, Microbiome, and Metabolites

The intestinal tract, comprised of small intestine, large intestine/colon, and rectum, is the central location for nutrient and water absorption. It harbors more than 10^13^ microorganisms, contains over 20 different hormones, and serves as the single largest immune compartment in the body (13, 59). Consequently, building and maintaining a homeostatic intestinal tract is a highly complex and broad concept that encompasses a multi-disciplinary approach including the immune system, host cells, gut microbiota and nutrients. Further complexity arises from the mutual interactions between the intestinal tract and other organ systems.

**Figure 2:**
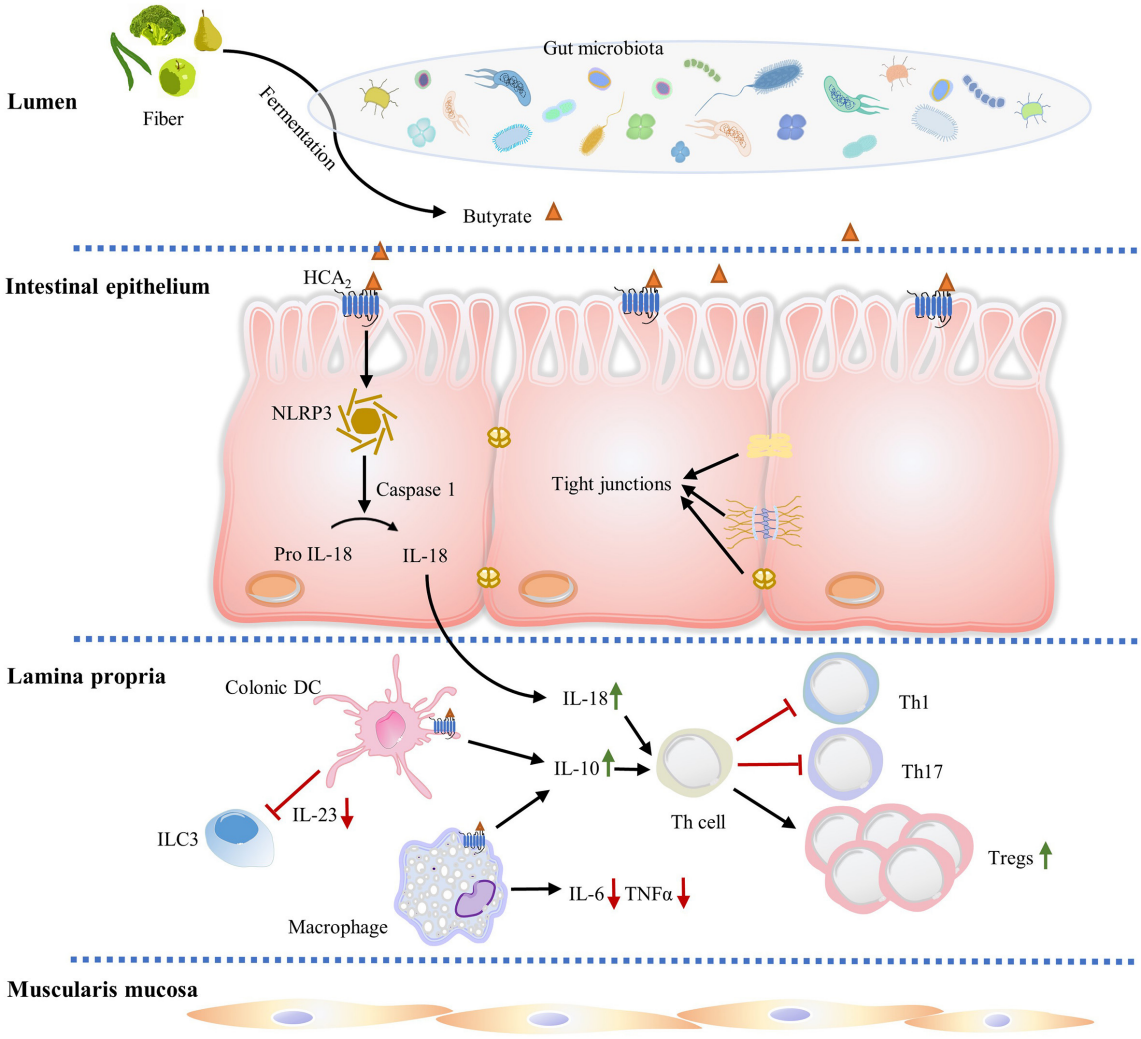
HCA_2_ regulates gut immune homeostasis. Butyrate produced by gut microbiota fermentation activates HCA_2_ expressed on IECs, macrophages, and dendritic cells. In IECs, HCA_2_ stimulation is associated with increased inflammasome activation, which processes pro-IL-18 into mature IL-18, which is critical in regulating mucosal immunity and epithelial integrity. IL-18 and ligand induced anti-inflammatory IL-10 (from intestinal macrophages and dendritic cells) promote naïve T cells differentiation and proliferation into immunomodulator Treg cells, which protect intestine from inflammation and colitis associated cancer. IL-18 and IL-10 also decrease the proinflammatory Th1 and Th17 cell number. In addition, HCA_2_ decreases proinflammatory cytokine IL-6 and TNF-α expression in intestinal macrophages and inhibits dendritic cell-induced IL-23 production to suppress ILC3-associated colonic inflammation.

The intestinal mucosa, a crucial site of innate and adaptive immune regulation, is comprised of IECs, lamina propria and muscularis mucosa (Figure 2). IECs are specialized epithelia comprised of many different cell types: epithelial stem cells which continuously self-renew by dividing and generate all differentiated intestinal cell types, enterocytes which absorb water and nutrients, goblet cells which secrete mucins to form a mucus layer boundary between the gut microbiota and host tissue, Paneth cells which secrete anti-microbial peptides, enteroendocrine cells which secrete hormones and cytokines capable of systemic or local effects, and microfold cells (M cells) which connect to the intestinal lymphoid follicles (60–63). The intestinal epithelium is bound together by tight junction proteins, which regulate the paracellular permeability and are essential for the integrity of the epithelial barrier. Tight junction proteins prevent harmful substances such as LPS, foreign antigens, toxins and microorganisms from entering into the blood stream (64).

IECs are well-equipped to recognize luminal pathogens by expressing different pattern recognition receptors, including NOD-like receptors (NLRs) in the cytosol and Toll-like receptors (TLRs) on the apical membrane and in endosomes, with the capacity to sample gram-positive and gram-negative infectious bacteria (65, 66). Additionally, various immune cells, including intraepithelial γδ-T cells and specialized mucosal macrophages, reside intercalated in the IEC layer, and function to sample pathogens from the lumen (67). IECs also express multimeric protein complexes known as inflammasomes that are important for intestinal immune homeostasis, inflammation, and tumorigenesis. Ligand stimulation of HCA_2_ expression is associated with increased NLRP3 inflammasome activation, which processes the proIL-18 into IL-18, an anti-inflammatory cytokine which is critical in regulating mucosal immunity and epithelial integrity (46) (Figure 2). Recent studies demonstrate that mice deficient in IL-18 have increased pathogenesis of colitis and colon cancer, and dysregulation of IL-1β expression exacerbates IBD (48, 68).

Immune cells are found in intestinal epithelium (intraepithelial lymphocytes) as well as in organized lymphoid tissues/organs, such as the Peyer's Patches (PPs) and mesenteric lymph nodes (MLNs). Substantial amounts of scattered innate and adaptive effector immune cells are also widely distributed in the lamina propria, which is a loosely packed connective tissue layer underlying the IEC layer (69–71) (Figure 2). Collectively, the lamina propria and IECs form a unique immunological compartment which contains the largest population of immune cells in the body, as well as supply the nerve, blood and lymph drainage for the entire mucosa (71). The lamina propria contains lymphocytes and numerous innate immune system-related cell populations, including eosinophils, macrophages, DCs, immunoglobulin (Ig) A secreting plasma cells, mast cells and innate lymphoid cells (ILCs) (71–73) (Figure 2). ILCs are a family of three innate effector cells (ILC1, ILC2, and ILC3) that are critical modulators of mucosal immunity (74). Particularly, ILC3 is implicated in innate intestinal inflammation though production of IFN-γ, IL-17, and IL-22 under induction by IL-1β and IL-23 (75). Depletion of ILC3 abrogates innate colitis, suggesting ILC3 is responsible for the intestinal pathogenesis (75). Bhatt et al. showed that HCA_2_ signaling limits IL-23 production by DCs, which further suppresses ILC3-mediated colonic inflammation (76) (Figure 2). Activation of HCA_2_ expressed on immune cells in colon lamina propria also modulates the frequency and number of Treg cells and IL-10 producing T cells (34) (Figure 2).

The gut microbiota is considered a commensal metabolic organ with critical roles in energy salvaging and nutrient absorption. It also functions in systemic immunity regulation and protection of the colonized host by eliminating pathogenic bacteria (77). Tan et al. compared fecal microbiota composition between WT and HCA_2_^−/−^ mice fed a high-fiber diet, and determined that loss of HCA_2_ alters microbiota composition dramatically (57). Specifically, HCA_2_^−/−^ mice show an increase of *Verrucomicrobiae, Alphaproteobacteria*, and *Bacilli*, and a decrease of *Bacteroidia* (57). Germ-free animals show extensive impaired maturation of isolated lymphoid follicles, PPs and MLNs, and are also defective in antibody production and cytokine secretion compared to conventional animals (78). The status of germ-free animals converts after colonization with normal gut microbiota, suggesting a dynamic relationship between the commensal organism and host immune system. Gut microbiota also plays an irreplaceable role in the regulation of host intestinal gene expression with around 700 genes altered remarkably in mice under germ-free conditions. Among them, the expression of *Hca*_2_ is reduced significantly in the ileum and colon under germ-free conditions, which is restored to normal levels after introduction of gut bacteria (35).

When the balance of gut microbiota ecosystems is disturbed (dysbiosis), tight junction barrier is compromised. Antigens, toxins and microorganisms can pass through the epithelium and trigger the immune response. Intestinal dysbiosis is commonly associated with a series of intestinal and extra-intestinal pathological disorders, including obesity, diabetes mellitus, multisystem organ failure, allergy, asthma, colitis-associated cancer and IBD (77, 79). Specifically, IBD patients shift their gut microbiota composition to an enrichment of *Desulfovibrio, Enterobacteriaceae, Ruminococcus gnavus*, and depletion of *Akkermansia Faecalibacterium prausnitzii*, and *Lachnospiraceae* (80).

Multiple evidence suggests that the composition of the intestinal microbiota can be altered by diet within hours to days, leading to aberrant immune responses (81–83). Extensive studies have demonstrated that the structure and function of the gut microbiota rapidly shifts and intestinal atrophy and low-grade inflammation occur under Western diets conditions within 1 day (84–89). Nevertheless, this influence is largely eliminated by manipulating the dietary fiber content in Western diets, allowing for protection against microbiota depletion, amelioration of the inflammation and restoration of colon length (84, 88). These beneficial aspects of fiber are largely attributed to bacterial fermentation products (SCFAs), including acetate, propionate and butyrate. SCFAs are sensed by specific immunomodulating receptors, including HCA_2_, GPR41, and GPR43, which are involved in intestinal immunoactivity, intestinal motility regulation and cytokine secretion (90).

Among the SCFAs, butyrate/HCA_2_-mediated signaling has received the most attention for its effects on intestinal homeostasis and may provide an important molecular link between gut bacteria and the host (91–94). Numerous studies have confirmed that antibiotic treatment causes gut microbiota dysbiosis by perturbing intestinal immune regulation, evidenced by a reduction in Treg cell numbers within the colon (95, 96). Niacin and HCA_2_ agonist supplementation efficiently rescues Treg cell depletion in antibiotic-treated WT mice, but this effect is nullified in HCA_2_^−/−^ mice (34). HCA_2_^−/−^ mice also show an inflammatory intestinal phenotype and enhanced susceptibility to azoxymethane (AOM) + dextran sulfate sodium (DSS)-induced colitis-associated colon cancer (34). Clinically, patients with ulcerative colitis and colitis-associated cancer suffer a remarkable depletion in the total amount of butyrate-producing bacteria in colon (97, 98), while irrigating the colon with butyrate significantly suppresses intestinal inflammation during ulcerative colitis (99). Hence, HCA_2_ is a critical link in the network of diet, microbiota, immune cells, and host cells which are necessary for the maintenance of intestinal homeostasis.

## Role of HCA_2_ in Intestinal Inflammation

The role of HCA_2_ in regulating intestinal immunological response and inflammation is multifaceted. Singh et al. found that the colons of mice lacking HCA_2_ present a unique status of CD4^+^ T cells, also known as T helper cells (Th cells) (34). These cells play an important role in immune regulation, where they mediate the activation of other immune cells though the release of various cytokines. Among the CD4^+^ T cells, Tregs express the transcription factor Forkhead box protein P3 (Foxp3), which is capable of potently suppressing immune responses. In colonic lamina propria of HCA_2_^−/−^ mice, the amount of Foxp3^+^/Treg cells among CD4^+^ T cells and anti-inflammatory IL-10 producing CD4^+^ T cells is significantly less than WT mice, while the frequency and number of CD4^+^ T cells producing the inflammatory cytokine IL-17 are increased (34). In contrast, a similar fraction of those cells distribute in splenic T cells from both WT and HCA_2_^−/−^ mice, suggesting that only the colon CD4^+^ T cells are specifically influenced by a lack of HCA_2_^−/−^ (34). Singh et al. reasoned that this proinflammatory phenotype of the HCA_2_^−/−^ mice colon is dependent on colonic DCs and macrophages, since they both express HCA_2_ and are critical inducers of naive T cell differentiation (100, 101). They addressed this by testing the ability of colonic DCs and macrophages from both WT and HCA_2_^−/−^ mice to induce differentiation of naive CD4^+^ T cells. As expected, HCA_2_^−/−^ colonic DCs and macrophages are defective in expression of retinaldehyde dehydrogenase 1 (RALDH1) and immunosuppressive cytokine IL-10, and express more proinflammatory cytokine IL-6 compared to WT DCs and macrophages. This change in expression leads naive CD4^+^ T cells to differentiate into proinflammatory Th17 cells, but not Treg cells and IL-10-producing CD4^+^ T cells (34). Likewise, HCA_2_^−/−^ is necessary to maintain normal anti-inflammatory IL-18 levels, as both mRNA and protein expression of IL-18 are significantly decreased in IECs of HCA_2_^−/−^ mice (34). Consistent with this evidence, Singh et al. also demonstrated that niacin treatment restored colonic Treg cell numbers in antibiotic-treated WT mice, and butyrate and niacin induced IL-10 and RALDH1 expression and promoted naïve T cells differentiation into Treg cells in macrophages and DCs in an HCA_2_-dependent manner (34) (Figure 2). In addition, butyrate and niacin increased expression of IL-18 in colonic epithelium of WT mice but not HCA_2_^−/−^ mice (34).

Bhatt et al. recently described another anti-inflammatory effect of HCA_2_ in restraining microbiota-induced IL-23 production to suppress ILC3-associated colonic inflammation (76). To diminish the influence of the adaptive immune system, they bred HCA_2_^−/−^ mice with recombination activating gene 1 (RAG1) deficient mice [no mature B and T lymphocytes (102)] to generate HCA_2_^−/−^ Rag1^−/−^ mice. HCA_2_^−/−^ Rag1^−/−^ mice spontaneously develop rectal prolapse and exhibit immune cell infiltration of the intestinal lamina propria, which is not seen in Rag1^−/−^ mice under the same conditions (76). In addition, colons of HCA_2_^−/−^ Rag1^−/−^ mice are larger and hypercellular, over proliferative with hyperchromatic and pseudostratified nuclei and have significantly elevated number of neutrophils compared to Rag1^−/−^ mice (76). As a result, HCA_2_^−/−^ Rag1^−/−^ mice have significantly higher colitis scores for colons and cecum compared to Rag1^−/−^ mice (76). Importantly, HCA_2_^−/−^ Rag1^−/−^ mice have significantly increased numbers of ILC3 in the colonic lamina propria, mesenteric lymph nodes and small intestine, leading to a markedly higher frequency of IL-17 in the colonic lamina propria and mesenteric lymph nodes (76). Niacin significantly decreases IL-23 production by colonic DCs and the numbers of ILC3 in Rag1^−/−^ mice, but fails to do so in HCA_2_^−/−^ Rag1^−/−^ mice (76). Furthermore, HCA_2_^−/−^ Rag1^−/−^ mice present signs of ongoing adenomatous transformation in the cecum and colonize a higher portion of IBD associated bacteria including *Bacteroidaceae, Porphyromonadaceae, Prevotellaceae, Streptococcaceae, Christensenellaceae, Mogibacteriaceae, Enterobacteriaceae*, and *Mycoplasmataceae*. Depletion of gut microbiota by antibiotics alleviates colonic inflammation by decreasing production of IL-23 and induction of ILC3 in HCA_2_^−/−^ Rag1^−/−^ mice (76).

Further detailed studies on the role of HCA_2_ in DSS-induced colitis treatment demonstrate that HCA_2_^−/−^ mice are highly susceptible to colitis development, with all experimental animals succumbing to death 10 days after DSS administration (3). In contrast, WT counterparts all survive through the entirety of the DSS treatment (34). Sodium butyrate markedly reduces inflammation and improves IECs barrier integrity by activating HCA_2_ signaling and suppressing the AKT-NF-κB p65 signaling pathway in 2,4,6-trinitrobenzene sulfonic acid (TNBS)-induced colitis, a model that resembles Crohn's disease (51, 52). In a similar study, a sodium butyrate-containing diet attenuates diarrhea symptoms and facilitates tight junction protein expression in the colon of piglets by acting on Akt signaling pathway in an HCA_2_-dependent manner (103). Another source of butyrate, tributyrin, is a chemically stable structured lipid that could be administered orally (104). Tributyrin supplementation prevents mice from chronic and acute ethanol-induced gut injury by improving gut barrier function (occludin, ZO-1) and increasing the expression of HCA_2_ in both ileum and proximal colon (105). In accordance with this, niacin administration attenuates iodoacetamide-induced colitis by a reduction in colon weight and colonic myeloperoxidase activity (a hallmark of colonic inflammation), and restores normal levels of colonic IL-10, tumor necrosis factor alpha (TNF-α), angiostatin and endostatin in a rat model (106). This beneficial effect of niacin is largely abolished by mepenzolate bromide, a HCA_2_ receptor blocker, indicating niacin/ HCA_2_ signaling ameliorates iodoacetamide-induced colitis (106). In addition to it oral pharmacologic activity, niacin is also a microbial-derived metabolite, produced by specific gut microbiota, including *Lactobacillus acidophilus, Bacteroides fragilis, Prevotella copri, Fusobacterium varium, Clostridium difficile, Bifidobacterium infantis*, and *Ruminococcus lactaris* (76, 107, 108). Niacin deficiency is associated with intestinal inflammation and diarrhea (76).

Overall, these reports provide compelling evidence that HCA_2_ signaling modulates immune cells to inhibit production of several inflammatory cytokines, pathways, and enzymes, leading to the suppression of experimental models of colitis.

## Role of HCA_2_ in Colon Cancer

**Figure 3:**
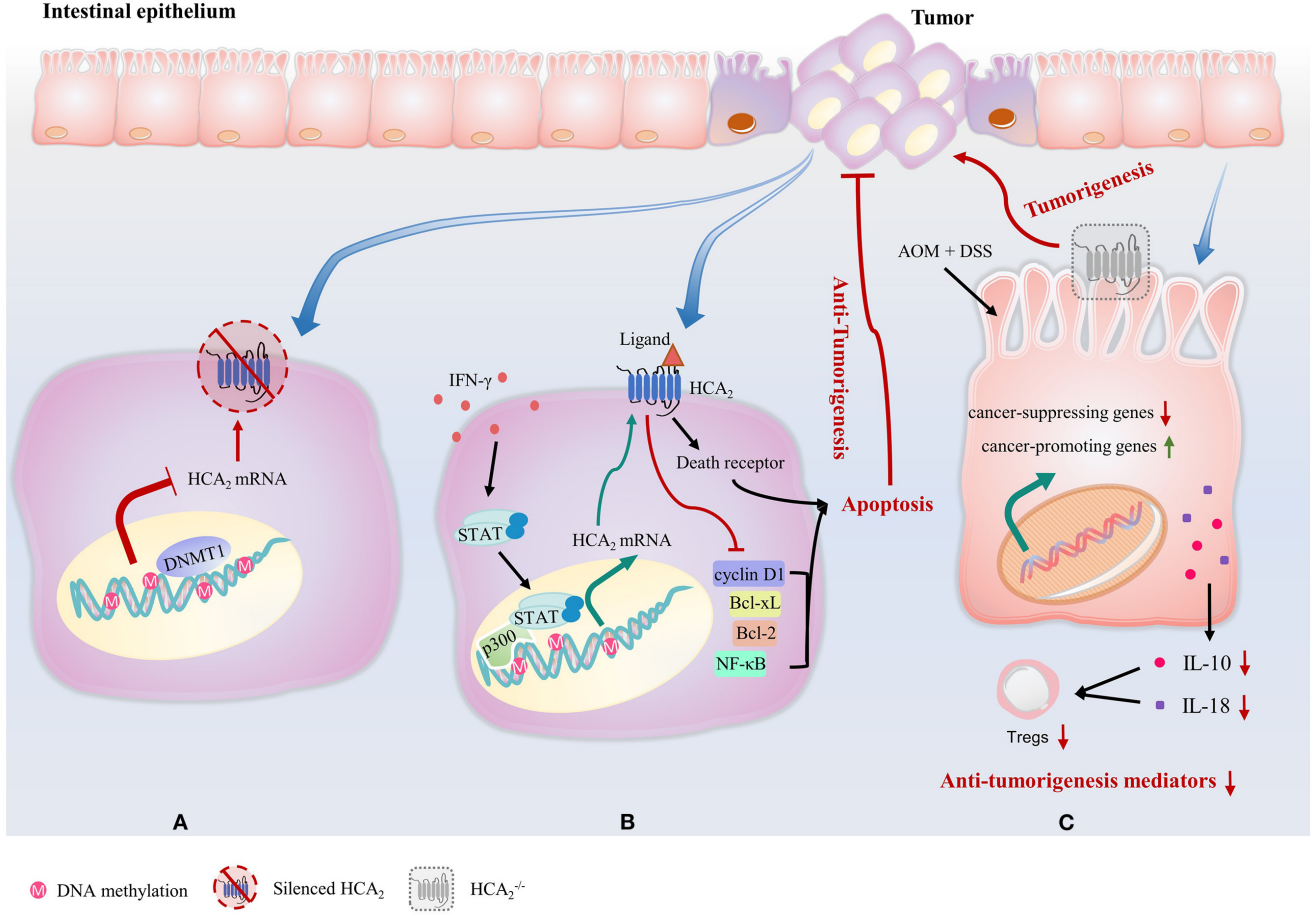
HCA_2_ plays a critical role in the suppression of colonic cancer. **(A)** Expression of HCA_2_ is silenced in colon cancer cells. This tumor-associated silencing of HCA_2_ involves DNMT1-mediated DNA methylation. **(B)** IFN-γ reverses DNA methylation-mediated HCA_2_ silencing without altering the methylation status of the HCA_2_ promoter. STAT1 is rapidly activated by IFN-γ and binds to the p300 promoter to activate p300 transcription, resulting in a permissive chromatin conformation at the HCA_2_ promoter to allow HCA_2_ transcription in colon carcinoma cells. Reexpression of HCA_2_ in cancer cell lines induces apoptosis by inhibiting of Bcl-2, Bcl-xL, cyclin D1, and NF-κB activity and upregulating the death receptor pathway in a ligand-dependent manner. **(C)** In mouse models of inflammation-associated colon cancer caused by AOM and DSS, the intestinal epithelium of HCA_2_^−/−^ mice display upregulated expression of colon cancer-promoting genes and decreased genes that inhibit colitis and colon carcinogenesis. HCA_2_^−/−^ mice also exhibit a severe impairment of IL-10 and IL-18 production, which leads to a decrease in Treg cell number.

HCA_2_ not only plays a critical role in the suppression of intestinal inflammation, but also has a significant effect on colonic cancer development and progression. Expression of HCA_2_ is silenced in colon cancer cell lines, and in both mice and humans with colon cancer (24). The tumor-associated silencing of HCA_2_ involves DNA methyltransferase 1 (DNMT1)-mediated DNA methylation (24) (Figure 3A). Reexpression of HCA_2_ in cancer cell lines induces apoptosis by inhibiting B-cell lymphoma (Bcl)-2, B-cell lymphoma-extra-large (Bcl-xL), cyclin D1 and NF-κB activity and upregulating the death receptor pathway in a ligand-dependent manner (Figure 3B). Butyrate is also an inhibitor of histone deacetylases, but this HCA_2_-mediated effort in colon cancer cells does not involve repressing histone deacetylation (24). Strikingly, Bardhan et al. discovered that IFN-γ reverses DNA methylation-mediated HCA_2_ silencing without altering the methylation status of the HCA_2_ promoter in colon carcinoma cells (109). Signal transducer and activator of transcription 1 (STAT1) is rapidly activated by IFN-γ and binds to the p300 promoter to activate p300 transcription. p300 is a histone acetyltransferase and a master transcriptional mediator in mammalian cells, resulting in a permissive chromatin conformation at the HCA_2_ promoter to allow STAT1 to activate HCA_2_ transcription despite DNA methylation (109) (Figure 3B).

Consistently, HCA_2_^−/−^ mice are more susceptible to the development of colon cancer (34, 46, 106, 110). In mouse models of inflammation-associated colon cancer caused by AOM and DSS, colons of HCA_2_^−/−^ mice shrink with highly increased myeloperoxidase activity, upregulated expression of colon cancer-promoting genes such as cyclin-D1, cyclin-B1, and cyclin-dependent kinase 1, decreased tight junction proteins expression and decreased expression of genes that inhibit colitis and colon carcinogenesis, such as transforming growth factor beta (*Tgfb)*1, *Tgfb2*, Solute Carrier Family 5 Member 8 (*Slc5a8*), MutS Homolog (*Msh*)2, and *Msh3* (34) (Figure 3C). In addition, HCA_2_^−/−^ mice exhibit a severe impairment of IL-10 and IL-18 production when compared to WT counterparts (34, 111) (Figure 3C). Histologically, crypt and epithelium structure damage, mucosa ulcerations and large amount of immune cell infiltration is observed in colons of AOM+DSS treated HCA_2_^−/−^ mice group, indicating epithelial barrier breakdown (34). Systemically, levels of both colonic and serum cytokines that promote colonic inflammation and carcinogenesis such as amyloid A, chemokine (C-X-C motif) ligand (CXCL) 1, C-C motif chemokine ligand (CCL) 2, IL-1β, IL-6, and IL-17 are all elevated. At the end of the AOM+DSS treatment regime, HCA_2_^−/−^ mice demonstrate anemia and increased number of large polyps on colon (34). Remarkably, niacin administration suppresses colon tumor development in antibiotic-treated microbiota-depleted WT mice (34). However, it also promotes colitis-associated cancer in HCA_2_^−/−^ mice, which is associated with an expansion of bacteria in *Prevotellaceae* family and TM7 phylum (34), suggesting microbiota/niacin protective effect is HCA_2_-dependent. In the same report, Singh et al. manipulated another mouse model of intestinal carcinogenesis, Apc^Min/+^, in which multiple intestinal neoplasia (*Min*) is a mutant allele of the murine adenomatous polyposis coli (*Apc*) locus (110). Apc^Min/+^ mice show significantly enlarged colonic polyp numbers, which were rescued by niacin treatment. However, niacin was not able to decrease the development of colonic polyps in HCA_2_^−/−^Apc^Min/+^ (34).

Taken together, these data demonstrate that HCA_2_ mediates cancer development and progression by promoting intestine mucosal immunity and decreasing cancer-promoting genes.

## Anti-Inflammatory Effects of HCA_2_ in Other Diseases

HCA_2_ signaling plays an essential role in preventing and reducing inflammation in the intestine. In addition, HCA_2_ has also been associated with anti-inflammatory effects in numerous disease states. In particular, various studies report that activation of HCA_2_ reduces inflammation in atherosclerosis (36), diabetes mellitus (25), diabetic retinopathy (31), neurodegenerative diseases (37, 38), sepsis (39), mammary cancer (40) and pancreatitis (41). Activation of HCA_2_ on immune cells in the vasculature by niacin reduces the progression of atherosclerosis and suppresses macrophage recruitment to atherosclerotic plaques (36). Chronic activation of HCA_2_ by niacin increases serum adiponectin in obese men with metabolic syndrome, suggesting a role in diabetes mellitus and obesity (112, 113). Additionally, in pancreatic islets of diabetic db/db mice as well as in type 2 diabetic (T2D) patients, HCA_2_ expression is decreased (114). Administration of GSK256073, a HCA_2_ agonist, notably reduced serum glucose and non-esterified fatty acids without inducing the niacin-associated side effect of cutaneous flushing in diabetic patients (25). In retinal pigmented epithelial cells, niacin-mediated activation of HCA_2_ suppresses TNF-α-induced NF-κB activation and IL-6 and monocyte chemoattractant protein-1 (MCP-1) secretion (31). HCA_2_ ligands have been also reported to attenuate inflammation in neurodegenerative diseases such as Parkinson's disease (115), Huntington's disease (38), Alzheimer's disease (116), multiple sclerosis (37), ischemic stroke (117) and traumatic brain injury (118), although, the mechanisms behind many of these beneficial effects have yet to be fully elucidated. In sepsis, niacin attenuated kidney and lung inflammation by decreasing NF-κB activation and subsequently decreasing inflammatory cytokines (39, 119, 120). As was the case in colon cancer, HCA_2_ functions as a tumor suppressor in mammary cancer via inhibition of genes involved in cell survival and anti-apoptotic pathways in human breast cancer cell lines (40). In pancreatitis, β-OHB supplementation inhibits macrophage NF-κB activation in an HCA_2_-dependent manner, and limits sterile inflammation (41). Moreover, HCA_2_ plays an antiviral role in reducing the Zika virus replication. HCA_2_ expression is significantly induced by Zika virus infection, while depletion of HCA_2_ resulted in significant increase of Zika virus RNA levels and viral yields, indicating that HCA_2_ can serve as a restriction factor for Zika virus and providing a potential target for anti- Zika virus therapeutic (121).

## Conclusion

There is mounting evidence summarized in this review that HCA_2_ plays an important role in modulating inflammation and carcinogenesis in the intestine. Ligands for the HCA_2_ receptor mediate a wide variety of inflammation-suppressing signaling events. NF-κB, NLRP3 and prostaglandins PGD_2_ and PGE_2_ have all been implicated as downstream targets of the HCA_2_ receptor, suggesting activation of one pathway may have beneficial or undesirable effects that are tissue-dependent. Therefore, tissue-specific, pharmacologic ligands which trigger bias signaling cascades, and therefore minimize less desirable downstream effects, are required. In addition, these pathways interweave the process of inflammatory and metabolic disorders through HCA_2_. Thus, this interplay of gut microbiota, HCA_2_ signaling and immune responses is a double-edged sword of inducing inflamed intestinal diseases or colon cancer and promoting intestinal homeostasis.

## Author Contributions

ZL was primarily responsible for researching and writing the manuscript (including the generation of figures). KM was responsible for writing specific sections and reviewing the manuscript. RJ proposed the topic of the review and supervised the writing and review of the manuscript. All authors contributed to the article and approved the submitted version.

## Conflict of Interest

The authors declare that the research was conducted in the absence of any commercial or financial relationships that could be construed as a potential conflict of interest.

## References

[1] Choct M. Managing gut health through nutrition. Br Poult Sci. (2009) 50:9–15. 10.1080/00071660802538632 19234925

[2] Abedi V Lu P Hontecillas R Verma M Vess G Philipson CW. Phase III placebo-controlled, randomized clinical trial with synthetic Crohn's disease patients to evaluate treatment response. Computational Modeling-Based Discovery of Novel Classes of Anti-Inflammatory Drugs that Target Lanthionine Synthetase C-Like Protein. Emerg Trends Comput Biol Bioinform Syst Biol Syst Appl. (2015) 2:169. 10.1016/b978-0-12-804203-8.00028-6

[3] Maloy KJ Powrie F. Intestinal homeostasis and its breakdown in inflammatory bowel disease. Nature. (2011) 474:298–306. 10.1038/nature10208 21677746

[4] Kaser A Lee A-H Franke A Glickman JN Zeissig S Tilg H. XBP1 links ER stress to intestinal inflammation and confers genetic risk for human inflammatory bowel disease. Cell. (2008) 134:743–56. 10.1016/j.cell.2008.07.021 18775308 PMC2586148

[5] Stokkers P Hommes D. New cytokine therapeutics for inflammatory bowel disease. Cytokine. (2004) 28:167–73. 10.1016/j.cyto.2004.07.012 15588691

[6] Guan Q Zhang J. Recent advances: the imbalance of cytokines in the pathogenesis of inflammatory bowel disease. Mediat Inflamm. (2017) 2017:4810258. 10.1155/2017/4810258 28420941 PMC5379128

[7] Feagins LA Souza RF Spechler SJ. Carcinogenesis in IBD: potential targets for the prevention of colorectal cancer. Nat Rev Gastroenterol Hepatol. (2009) 6:297–305. 10.1038/nrgastro.2009.44 19404270

[8] Kinugasa T Akagi Y. Status of colitis-associated cancer in ulcerative colitis. World J Gastrointest Oncol. (2016) 8:351. 10.4251/wjgo.v8.i4.351 27096030 PMC4824713

[9] Sairenji T Collins KL Evans DV. An update on inflammatory bowel disease. Prim Care. (2017) 44:673–692. 10.1016/j.pop.2017.07.010 29132528

[10] Dahlhamer JM Zammitti EP Ward BW Wheaton AG Croft JB. Prevalence of inflammatory bowel disease among adults aged≥ 18 years—United States, 2015. Morbid Mortal Week Rep. (2016) 65:1166–9. 10.15585/mmwr.mm6542a3 27787492

[11] Monteleone G Fina D Caruso R Pallone F. New mediators of immunity and inflammation in inflammatory bowel disease. Curr Opin Gastroenterol. (2006) 22:361–4. 10.1097/01.mog.0000231808.10773.8e 16760750

[12] Guan Q. A comprehensive review and update on the pathogenesis of inflammatory bowel disease. J Immunol Res. (2019) 2019:7247238. 10.1155/2019/7247238 31886308 PMC6914932

[13] Kraehenbuhl J-P Neutra MR. Molecular and cellular basis of immune protection of mucosal surfaces. Physiol Rev. (1992) 72:853–79. 10.1152/physrev.1992.72.4.853 1438580

[14] Nomura H Nielsen BW Matsushima K. Molecular cloning of cDNAs encoding a LD78 receptor and putative leukocyte chemotactic peptide receptors. Int Immunol. (1993) 5:1239–49. 10.1093/intimm/5.10.1239 7505609

[15] Gille A Bodor ET Ahmed K Offermanns S. Nicotinic acid: pharmacological effects and mechanisms of action. Annu Rev Pharmacol Toxicol. (2008) 48:79–106. 10.1146/annurev.pharmtox.48.113006.094746 17705685

[16] Schaub A Fütterer A Pfeffer K. PUMA-G, an IFN-γ-inducible gene in macrophages is a novel member of the seven transmembrane spanning receptor superfamily. Eur J Immunol. (2001) 31:3714–25. 10.1002/1521-4141(200112)31:12<3714::AID-IMMU3714>3.0.CO;2-1 11745392

[17] Soga T Kamohara M Takasaki J Matsumoto S-I Saito T Ohishi T. Molecular identification of nicotinic acid receptor. Biochem Biophys Res Commun. (2003) 303:364–9. 10.1016/S0006-291X(03)00342-5 12646212

[18] Tunaru S Kero J Schaub A Wufka C Blaukat A Pfeffer K. PUMA-G and HM74 are receptors for nicotinic acid and mediate its anti-lipolytic effect. Nat Med. (2003) 9:352. 10.1038/nm824 12563315

[19] Wise A Foord SM Fraser NJ Barnes AA Elshourbagy N Eilert M. Molecular identification of high and low affinity receptors for nicotinic acid. J Biol Chem. (2003) 278:9869–74. 10.1074/jbc.M210695200 12522134

[20] Benyó Z Gille A Kero J Csiky M Suchánková MC Nüsing RM. GPR109A (PUMA-G/HM74A) mediates nicotinic acid–induced flushing. J Clin Investig. (2005) 115:3634–40. 10.1172/JCI23626 16322797 PMC1297235

[21] Hanson J Gille A Zwykiel S Lukasova M Clausen BE Ahmed K. Nicotinic acid–and monomethyl fumarate–induced flushing involves GPR109A expressed by keratinocytes and COX-2–dependent prostanoid formation in mice. J Clin Investig. (2010) 120:2910–9. 10.1172/JCI42273 20664170 PMC2912194

[22] Taggart AK Kero J Gan X Cai T-Q Cheng K Ippolito M. (D)-β-hydroxybutyrate inhibits adipocyte lipolysis via the nicotinic acid receptor PUMA-G. J Biol Chem. (2005) 280:26649–52. 10.1074/jbc.C500213200 15929991

[23] Offermanns S Colletti SL Lovenberg TW Semple G Wise A IJzerman AP. International Union of Basic and Clinical Pharmacology. LXXXII: nomenclature and classification of hydroxy-carboxylic acid receptors (GPR81, GPR109A, and GPR109B). Pharmacol Rev. (2011) 63:269–90. 10.1124/pr.110.003301 21454438

[24] Thangaraju M Cresci GA Liu K Ananth S Gnanaprakasam JP Browning DD. GPR109A is a G-protein–coupled receptor for the bacterial fermentation product butyrate and functions as a tumor suppressor in colon. Cancer Res. (2009) 69:2826–32. 10.1158/0008-5472.CAN-08-4466 19276343 PMC3747834

[25] Dobbins R Shearn S Byerly R Gao F Mahar K Napolitano A. GSK256073, a selective agonist of G-protein coupled receptor 109A (GPR109A) reduces serum glucose in subjects with type 2 diabetes mellitus. Diabetes Obes Metab. (2013) 15:1013–21. 10.1111/dom.12132 23701262

[26] Ahmed K Tunaru S Offermanns S. GPR109A, GPR109B and GPR81, a family of hydroxy-carboxylic acid receptors. Trends Pharmacol Sci. (2009) 30:557–62. 10.1016/j.tips.2009.09.001 19837462

[27] Semple G Skinner PJ Gharbaoui T Shin Y-J Jung J-K Cherrier MC. 3-(1 H-tetrazol-5-yl)-1, 4, 5, 6-tetrahydro-cyclopentapyrazole (MK-0354): a partial agonist of the nicotinic acid receptor, G-protein coupled receptor 109a, with antilipolytic but no vasodilatory activity in mice. J Med Chem. (2008) 51:5101–8. 10.1021/jm800258p 18665582

[28] Maciejewski-Lenoir D Richman JG Hakak Y Gaidarov I Behan DP Connolly DT. Langerhans cells release prostaglandin D2 in response to nicotinic acid. J Investig Dermatol. (2006) 126:2637–46. 10.1038/sj.jid.5700586 17008871

[29] Yousefi S Cooper PR Mueck B Potter SL Jarai G., cDNA representational difference analysis of human neutrophils stimulated by GM-CSF. Biochem Biophys Res Commun. (2000) 277:401–9. 10.1006/bbrc.2000.3678 11032736

[30] Shi Y Lai X Ye L Chen K Cao Z Gong W. Activated niacin receptor HCA2 inhibits chemoattractant-mediated macrophage migration via Gβγ/PKC/ERK1/2 pathway and heterologous receptor desensitization. Sci Rep. (2017) 7:42279. 10.1038/srep42279 28186140 PMC5301212

[31] Gambhir D Ananth S Veeranan-Karmegam R Elangovan S Hester S Jennings E. GPR109A as an anti-inflammatory receptor in retinal pigment epithelial cells and its relevance to diabetic retinopathy. Investig Ophthalmol Vis Sci. (2012) 53:2208–17. 10.1167/iovs.11-8447 22427566 PMC4627510

[32] Graff E Norris O Sandey M Kemppainen R Judd R. Characterization of the hydroxycarboxylic acid receptor 2 in cats. Domest Anim Endocrinol. (2015) 53:88–94. 10.1016/j.domaniend.2015.06.001 26164006

[33] Ge H Weiszmann J Reagan JD Gupte J Baribault H Gyuris T. Elucidation of signaling and functional activities of an orphan GPCR, GPR81. J Lipid Res. (2008) 49:797–803. 10.1194/jlr.M700513-JLR200 18174606

[34] Singh N Gurav A Sivaprakasam S Brady E Padia R Shi H. Activation of Gpr109a, receptor for niacin and the commensal metabolite butyrate, suppresses colonic inflammation and carcinogenesis. Immunity. (2014) 40:128–39. 10.1016/j.immuni.2013.12.007 24412617 PMC4305274

[35] Cresci GA Thangaraju M Mellinger JD Liu K Ganapathy V. Colonic gene expression in conventional and germ-free mice with a focus on the butyrate receptor GPR109A and the butyrate transporter SLC5A8. J Gastrointest Surg. (2010) 14:449–61. 10.1007/s11605-009-1045-x 20033346

[36] Lukasova M Malaval C Gille A Kero J Offermanns S. Nicotinic acid inhibits progression of atherosclerosis in mice through its receptor GPR109A expressed by immune cells. J Clin Investig. (2011) 121:1163–73. 10.1172/JCI41651 21317532 PMC3048854

[37] Hao J Liu R Turner G Shi F-D Rho JM. Inflammation-mediated memory dysfunction and effects of a ketogenic diet in a murine model of multiple sclerosis. PLoS ONE. (2012) 7:e35476. 10.1371/journal.pone.0035476 22567104 PMC3342287

[38] Lim S Chesser AS Grima JC Rappold PM Blum D Przedborski S. D-β-hydroxybutyrate is protective in mouse models of Huntington's disease. PLoS ONE. (2011) 6:e24620. 10.1371/journal.pone.0024620 21931779 PMC3171454

[39] Kwon WY Suh GJ Kim KS Kwak YH. Niacin attenuates lung inflammation and improves survival during sepsis by downregulating the nuclear factor-κB pathway. Crit Care Med. (2011) 39:328–34. 10.1097/CCM.0b013e3181feeae4 20975550

[40] Elangovan S Pathania R Ramachandran S Ananth S Padia RN Lan L. The niacin/butyrate receptor GPR109A suppresses mammary tumorigenesis by inhibiting cell survival. Cancer Res. (2014) 74:1166–78. 10.1158/0008-5472.CAN-13-1451 24371223 PMC3944627

[41] Hoque R Mehal WZ. Inflammasomes in pancreatic physiology and disease. Am J Physiol Gastrointest Liver Physiol. (2015) 308:G643–51. 10.1152/ajpgi.00388.2014 25700081 PMC4398840

[42] Carlson LA Orö L. The effect of nicotinic acid on the plasma free fatty acids demonstration of a metabolic type of sympathicolysis. Acta Med Scand. (1962) 172:641–5. 10.1111/j.0954-6820.1962.tb07203.x 14018702

[43] Hanson J Gille A Offermanns S. Role of HCA2 (GPR109A) in nicotinic acid and fumaric acid ester-induced effects on the skin. Pharmacol Ther. (2012) 136:1–7. 10.1016/j.pharmthera.2012.06.003 22743741

[44] Ye L Cao Z Lai X Shi Y Zhou N. Niacin ameliorates hepatic steatosis by inhibiting de novo lipogenesis via a GPR109A-mediated PKC–ERK1/2–AMPK signaling pathway in C57BL/6 mice fed a high-fat diet. J Nutr. (2020) 150:672–84. 10.1093/jn/nxz303 31858105

[45] Ye L Cao Z Lai X Wang W Guo Z Yan L. Niacin fine-tunes energy homeostasis through canonical GPR109A signaling. FASEB J. (2019) 33:4765–79. 10.1096/fj.201801951R 30596513

[46] Macia L Tan J Vieira AT Leach K Stanley D Luong S. Metabolite-sensing receptors GPR43 and GPR109A facilitate dietary fibre-induced gut homeostasis through regulation of the inflammasome. Nat Commun. (2015) 6:6734. 10.1038/ncomms7734 25828455

[47] Zaki MH Lamkanfi M Kanneganti T-D. The Nlrp3 inflammasome: contributions to intestinal homeostasis. Trends Immunol. (2011) 32:171–9. 10.1016/j.it.2011.02.002 21388882 PMC3070791

[48] Rathinam VA Fitzgerald KA. Inflammasome complexes: emerging mechanisms and effector functions. Cell. (2016) 165:792–800. 10.1016/j.cell.2016.03.046 27153493 PMC5503689

[49] Gautam J Banskota S Shah S Jee JG Kwon E Wang Y. 4-Hydroxynonenal-induced GPR109A (HCA2 receptor) activation elicits bipolar responses, Gαi-mediated anti-inflammatory effects and Gβγ-mediated cell death. Br J Pharmacol. (2018) 175:2581–98. 10.1111/bph.14174 29473951 PMC6003634

[50] Offermanns S Schwaninger M. Nutritional or pharmacological activation of HCA2 ameliorates neuroinflammation. Trends Mol Med. (2015) 21:245–55. 10.1016/j.molmed.2015.02.002 25766751

[51] Zandi-Nejad K Takakura A Jurewicz M Chandraker AK Offermanns S Mount D. The role of HCA2 (GPR109A) in regulating macrophage function. FASEB J. (2013) 27:4366–74. 10.1096/fj.12-223933 23882124 PMC3804742

[52] Fu S-P Wang J-F Xue W-J Liu H-M Liu B-R Zeng Y-L. Anti-inflammatory effects of BHBA in both *in vivo* and *in vitro* Parkinson's disease models are mediated by GPR109A-dependent mechanisms. J Neuroinflamm. (2015) 12:1–14. 10.1186/s12974-014-0230-3 25595674 PMC4310035

[53] Fu S-P Li S-N Wang J-F Li Y Xie S-S Xue W-J. BHBA suppresses LPS-induced inflammation in BV-2 cells by inhibiting NF-κ B activation. Mediat Inflamm. (2014) 2014:983401. 10.1155/2014/983401 24803746 PMC3997897

[54] Digby JE Martinez F Jefferson A Ruparelia N Chai J Wamil M. Anti-inflammatory effects of nicotinic acid in human monocytes are mediated by GPR109A dependent mechanisms. Arterioscler Thromb Vasc Biol. (2012) 32:669–76. 10.1161/ATVBAHA.111.241836 22267479 PMC3392598

[55] Vitali Mingozzi F Broggi A Barresi S Zolezzi F Bayry J. Migratory, not lymphoid-resident, dendritic cells maintain peripheral self-tolerance and prevent autoimmunity via induction of iTreg cells. Blood. (2012) 120:1237–45. 10.1182/blood-2011-09-379776 22760781

[56] Bakdash G Vogelpoel LT Van Capel TM Kapsenberg ML de Jong EC. Retinoic acid primes human dendritic cells to induce gut-homing, IL-10-producing regulatory T cells. Mucosal Immunol. (2015) 8:265–78. 10.1038/mi.2014.64 25027601

[57] Tan J McKenzie C Vuillermin PJ Goverse G Vinuesa CG Mebius RE. Dietary fiber and bacterial SCFA enhance oral tolerance and protect against food allergy through diverse cellular pathways. Cell Rep. (2016) 15:2809–24. 10.1016/j.celrep.2016.05.047 27332875

[58] Kostylina G Simon D Fey M Yousefi S Simon H-U. Neutrophil apoptosis mediated by nicotinic acid receptors (GPR109A). Cell Death Differ. (2008) 15:134–42. 10.1038/sj.cdd.4402238 17932499

[59] Arumugam M Raes J Pelletier E Le Paslier D Yamada T Mende DR. Enterotypes of the human gut microbiome. Nature. (2011) 473:174–80. 10.1038/nature09944 21508958 PMC3728647

[60] Karaki S-I Mitsui R Hayashi H Kato I Sugiya H Iwanaga T. Short-chain fatty acid receptor, GPR43, is expressed by enteroendocrine cells and mucosal mast cells in rat intestine. Cell Tissue Res. (2006) 324:353–60. 10.1007/s00441-005-0140-x 16453106

[61] Weiser MM. Intestinal epithelial cell surface membrane glycoprotein synthesis I. An indicator of cellular differentiation. J Biol Chem. (1973) 248:2536–41. 10.1016/S0021-9258(19)44141-0 4698230

[62] Peterson LW Artis D. Intestinal epithelial cells: regulators of barrier function and immune homeostasis. Nat Rev Immunol. (2014) 14:141–53. 10.1038/nri3608 24566914

[63] Umar S. Intestinal stem cells. Curr Gastroenterol Rep. (2010) 12:340–8. 10.1007/s11894-010-0130-3 20683682 PMC2965634

[64] Gonzalez-Mariscal L Betanzos A Nava P Jaramillo B. Tight junction proteins. Progr Biophys Mol Biol. (2003) 81:1–44. 10.1016/S0079-6107(02)00037-8 12475568

[65] Hisamatsu T Suzuki M Reinecker H-C Nadeau WJ McCormick BA Podolsky DK. CARD15/NOD2 functions as an antibacterial factor in human intestinal epithelial cells. Gastroenterology. (2003) 124:993–1000. 10.1053/gast.2003.50153 12671896

[66] Abreu MT. Toll-like receptor signalling in the intestinal epithelium: how bacterial recognition shapes intestinal function. Nat Rev Immunol. (2010) 10:131–44. 10.1038/nri2707 20098461

[67] Chen Y Chou K Fuchs E Havran WL Boismenu R. Protection of the intestinal mucosa by intraepithelial γδ T cells. Proc Natl Acad Sci USA. (2002) 99:14338–43. 10.1073/pnas.212290499 12376619 PMC137885

[68] Zaki MH Boyd KL Vogel P Kastan MB Lamkanfi M Kanneganti T-D. The NLRP3 inflammasome protects against loss of epithelial integrity and mortality during experimental colitis. Immunity. (2010) 32:379–91. 10.1016/j.immuni.2010.03.003 20303296 PMC2982187

[69] Niess JH Brand S Gu X Landsman L Jung S McCormick BA. CX3CR1-mediated dendritic cell access to the intestinal lumen and bacterial clearance. Science. (2005) 307:254–8. 10.1126/science.1102901 15653504

[70] Varol C Vallon-Eberhard A Elinav E Aychek T Shapira Y Luche H. Intestinal lamina propria dendritic cell subsets have different origin and functions. Immunity. (2009) 31:502–12. 10.1016/j.immuni.2009.06.025 19733097

[71] Mowat AM Agace WW. Regional specialization within the intestinal immune system. Nat Rev Immunol. (2014) 14:667–85. 10.1038/nri3738 25234148

[72] Hooper LV Macpherson AJ. Immune adaptations that maintain homeostasis with the intestinal microbiota. Nat Rev Immunol. (2010) 10:159–69. 10.1038/nri2710 20182457

[73] Bernink JH Krabbendam L Germar K de Jong E Gronke K Kofoed-Nielsen M. Interleukin-12 and-23 control plasticity of CD127+ group 1 and group 3 innate lymphoid cells in the intestinal lamina propria. Immunity. (2015) 43:146–60. 10.1016/j.immuni.2015.06.019 26187413

[74] Artis D Spits H. The biology of innate lymphoid cells. Nature. (2015) 517:293–301. 10.1038/nature14189 25592534

[75] Buonocore S Ahern PP Uhlig HH Ivanov II Littman DR Maloy KJ. Innate lymphoid cells drive interleukin-23-dependent innate intestinal pathology. Nature. (2010) 464:1371–5. 10.1038/nature08949 20393462 PMC3796764

[76] Bhatt B Zeng P Zhu H Sivaprakasam S Li S Xiao H. Gpr109a limits microbiota-induced IL-23 production to constrain ILC3-mediated colonic inflammation. J Immunol. (2018)200:2905–14. 10.4049/jimmunol.1701625 29514953 PMC5893356

[77] Guarner F Malagelada J-R. Gut flora in health and disease. Lancet. (2003) 361:512–9. 10.1016/S0140-6736(03)12489-0 12583961

[78] Round JL Mazmanian SK. The gut microbiota shapes intestinal immune responses during health and disease. Nat Rev Immunol. (2009) 9:313–23. 10.1038/nri2515 19343057 PMC4095778

[79] Carding S Verbeke K Vipond DT Corfe BM Owen LJ. Dysbiosis of the gut microbiota in disease. Microb Ecol Health Dis. (2015) 26:26191. 10.3402/mehd.v26.26191 25651997 PMC4315779

[80] Berry D Reinisch W. Intestinal microbiota: a source of novel biomarkers in inflammatory bowel diseases? Best practice & research. Clin Gastroenterol. (2013) 27:47–58. 10.1016/j.bpg.2013.03.005 23768552

[81] Brown K DeCoffe D Molcan E Gibson DL. Diet-induced dysbiosis of the intestinal microbiota and the effects on immunity and disease. Nutrients. (2012) 4:1095–119. 10.3390/nu4081095 23016134 PMC3448089

[82] Howe A Ringus DL Williams RJ Choo Z-N Greenwald SM Owens SM. Divergent responses of viral and bacterial communities in the gut microbiome to dietary disturbances in mice. ISME J. (2016) 10:1217–27. 10.1038/ismej.2015.183 26473721 PMC5029215

[83] Cummings J Jenkins D Wiggins H. Measurement of the mean transit time of dietary residue through the human gut. Gut. (1976) 17:210–8. 10.1136/gut.17.3.210 1269989 PMC1411154

[84] Chassaing B Miles-Brown J Pellizzon M Ulman E Ricci M Zhang L. Lack of soluble fiber drives diet-induced adiposity in mice. Am J Physiol Gastrointest Liver Physiol. (2015) 309:G528–41. 10.1152/ajpgi.00172.2015 26185332 PMC4593822

[85] Alou MT Lagier J-C Raoult D. Diet influence on the gut microbiota and dysbiosis related to nutritional disorders. Hum Microbiome J. (2016) 1:3–11. 10.1016/j.humic.2016.09.001

[86] Buford TW. (Dis) Trust your gut: the gut microbiome in age-related inflammation, health, and disease. Microbiome. (2017) 5:80. 10.1186/s40168-017-0296-0 28709450 PMC5512975

[87] Solas M Milagro FI Ramírez MJ Martínez JA. Inflammation and gut-brain axis link obesity to cognitive dysfunction: plausible pharmacological interventions. Curr Opin Pharmacol. (2017) 37:87–92. 10.1016/j.coph.2017.10.005 29107872

[88] Zou J Chassaing B Singh V Pellizzon M Ricci M Fythe MD. Fiber-mediated nourishment of gut microbiota protects against diet-induced obesity by restoring IL-22-mediated colonic health. Cell Host Microbe. (2018) 23:41–53. e44. 10.1016/j.chom.2017.11.003 29276170 PMC6005180

[89] Martinez KB Leone V Chang EB. Western diets, gut dysbiosis, and metabolic diseases: are they linked? Gut Microbes. (2017) 8:130–42. 10.1080/19490976.2016.1270811 28059614 PMC5390820

[90] Priyadarshini M Kotlo KU Dudeja PK Layden BT. Role of short chain fatty acid receptors in intestinal physiology and pathophysiology. Compr Physiol. (2018) 8:1091–115. 10.1002/cphy.c170050 29978895 PMC6058973

[91] Mukhopadhya I Segal JP Carding SR Hart AL Hold GL. The gut virome: the ‘missing link'between gut bacteria and host immunity? Ther Adv Gastroenterol. (2019) 12:1756284819836620. 10.1177/1756284819836620 30936943 PMC6435874

[92] Rooks MG Garrett WS. Gut microbiota, metabolites and host immunity. Nat Rev Immunol. (2016) 16:341–52. 10.1038/nri.2016.42 27231050 PMC5541232

[93] Levy M Blacher E Elinav E. Microbiome, metabolites and host immunity. Curr Opin Microbiol. (2017) 35:8–15. 10.1016/j.mib.2016.10.003 27883933

[94] Kelly D Conway S Aminov R. Commensal gut bacteria: mechanisms of immune modulation. Trends Immunol. (2005) 26:326–33. 10.1016/j.it.2005.04.008 15922949

[95] Atarashi K Tanoue T Shima T Imaoka A Kuwahara T Momose Y. Induction of colonic regulatory T cells by indigenous Clostridium species. Science. (2011) 331:337–41. 10.1126/science.1198469 21205640 PMC3969237

[96] Smith PM Howitt MR Panikov N Michaud M Gallini CA Bohlooly-Y M. The microbial metabolites, short-chain fatty acids, regulate colonic Treg cell homeostasis. Science. (2013) 341:569–73. 10.1126/science.1241165 23828891 PMC3807819

[97] Frank DN Amand ALS Feldman RA Boedeker EC Harpaz N Pace NR. Molecular-phylogenetic characterization of microbial community imbalances in human inflammatory bowel diseases. Proc Natl Acad Sci USA. (2007) 104:13780–85. 10.1073/pnas.0706625104 17699621 PMC1959459

[98] Wang T Cai G Qiu Y Fei N Zhang M Pang X. Structural segregation of gut microbiota between colorectal cancer patients and healthy volunteers. ISME J. (2012) 6:320–9. 10.1038/ismej.2011.109 21850056 PMC3260502

[99] Hamer HM Jonkers D Venema K Vanhoutvin S Troost F Brummer RJ. The role of butyrate on colonic function. Alim Pharmacol Ther. (2008) 27:104–19. 10.1111/j.1365-2036.2007.03562.x 17973645

[100] Coombes JL Siddiqui KR Arancibia-Cárcamo CV Hall J Sun C-M Belkaid Y. A functionally specialized population of mucosal CD103+ DCs induces Foxp3+ regulatory T cells via a TGF-β-and retinoic acid–dependent mechanism. J Exp Med. (2007) 204:1757–64. 10.1084/jem.20070590 17620361 PMC2118683

[101] Manicassamy S Reizis B Ravindran R Nakaya H Salazar-Gonzalez RM Wang Y-C. Activation of β-catenin in dendritic cells regulates immunity vs. tolerance in the intestine. Science. (2010) 329:849–53. 10.1126/science.1188510 20705860 PMC3732486

[102] Mombaerts P Iacomini J Johnson RS Herrup K Tonegawa S Papaioannou VE. RAG-1-deficient mice have no mature B and T lymphocytes. Cell. (1992) 68:869–77. 10.1016/0092-8674(92)90030-G 1547488

[103] Feng W Wu Y Chen G Fu S Li B Huang B. Sodium butyrate attenuates diarrhea in weaned piglets and promotes tight junction protein expression in colon in a GPR109A-dependent manner. Cell Physiol Biochem. (2018) 47:1617–29. 10.1159/000490981 29949795

[104] Wächtershäuser A Stein J. Rationale for the luminal provision of butyrate in intestinal diseases. Eur J Nutr. (2000) 39:164–71. 10.1007/s003940070020 11079736

[105] Cresci GA Bush K Nagy LE. Tributyrin supplementation protects mice from acute ethanol-induced gut injury. Alcohol Clin Exp Res. (2014) 38:1489–501. 10.1111/acer.12428 24890666 PMC4185400

[106] Salem HA Wadie W. Effect of niacin on inflammation and angiogenesis in a murine model of ulcerative colitis. Sci Rep. (2017) 7:1–8. 10.1038/s41598-017-07280-y 28769047 PMC5541000

[107] Kurnasov O Goral V Colabroy K Gerdes S Anantha S Osterman A. NAD biosynthesis: identification of the tryptophan to quinolinate pathway in bacteria. Chem Biol. (2003) 10:1195–204. 10.1016/j.chembiol.2003.11.011 14700627

[108] Gazzaniga F Stebbins R Chang SZ McPeek MA Brenner C. Microbial NAD metabolism: lessons from comparative genomics. Microbiol Mol Biol Rev. (2009) 73:529–41. 10.1128/MMBR.00042-08 19721089 PMC2738131

[109] Bardhan K Paschall AV Yang D Chen MR Simon PS Bhutia YD. IFNγ induces DNA methylation–silenced GPR109A expression via pSTAT1/p300 and H3K18 acetylation in colon cancer. Cancer Immunol Res. (2015) 3:795–805. 10.1158/2326-6066.CIR-14-0164 25735954 PMC4491007

[110] Li Y Kundu P Seow SW de Matos CT Aronsson L Chin KC. Gut microbiota accelerate tumor growth via c-jun and STAT3 phosphorylation in APC Min/+ mice. Carcinogenesis. (2012) 33:1231–8. 10.1093/carcin/bgs137 22461519

[111] Offermanns S. Hydroxy-carboxylic acid receptor actions in metabolism. Trends Endocrinol Metab. (2017) 28:227–36. 10.1016/j.tem.2016.11.007 28087125

[112] Plaisance EP Lukasova M Offermanns S Zhang Y Cao G Judd RL. Niacin stimulates adiponectin secretion through the GPR109A receptor. Am J Physiol Endocrinol Metab. (2009) 296:E549–58. 10.1152/ajpendo.91004.2008 19141678

[113] Plaisance EP Grandjean PW Brunson BL Judd RL. Increased total and high–molecular weight adiponectin after extended-release niacin. Metabolism. (2008) 57:404–9. 10.1016/j.metabol.2007.10.018 18249215

[114] Wang N Guo D-Y Tian X Lin H-P Li Y-P Chen S-J. Niacin receptor GPR109A inhibits insulin secretion and is down-regulated in type 2 diabetic islet beta-cells. Gen Comp Endocrinol. (2016) 237:98–108. 10.1016/j.ygcen.2016.08.011 27570060

[115] Tieu K Perier C Caspersen C Teismann P Wu D-C Yan S-D. D-β-Hydroxybutyrate rescues mitochondrial respiration and mitigates features of Parkinson disease. J Clin Investig. (2003) 112:892–901. 10.1172/JCI200318797 12975474 PMC193668

[116] Van der Auwera I Wera S Van Leuven F Henderson ST. A ketogenic diet reduces amyloid beta 40 and 42 in a mouse model of Alzheimer's disease. Nutr Metab. (2005) 2:28. 10.1186/1743-7075-2-28 16229744 PMC1282589

[117] Puchowicz MA Zechel JL Valerio J Emancipator DS Xu K Pundik S. Neuroprotection in diet-induced ketotic rat brain after focal ischemia. J Cerebr Blood Flow Metab. (2008) 28:1907–16. 10.1038/jcbfm.2008.79 18648382 PMC3621146

[118] Hu Z-G Wang H-D Qiao L Yan W Tan Q-F Yin H-X. The protective effect of the ketogenic diet on traumatic brain injury-induced cell death in juvenile rats. Brain Injury. (2009) 23:459–65. 10.1080/02699050902788469 19408168

[119] Gurujeyalakshmi G Wang Y Giri SN. Taurine and niacin block lung injury and fibrosis by down-regulating bleomycin-induced activation of transcription nuclear factor-κB in mice. J Pharmacol Exp Ther. (2000) 293:82–90. 10734156

[120] Cho K-H Kim H-J Rodriguez-Iturbe B Vaziri ND. Niacin ameliorates oxidative stress, inflammation, proteinuria, and hypertension in rats with chronic renal failure. Am J Physiol Renal Physiol. (2009) 297:F106–13. 10.1152/ajprenal.00126.2009 19420110

[121] Ma X Luo X Zhou S Huang Y Chen C Huang C. Hydroxycarboxylic acid receptor 2 is a Zika virus restriction factor that can be induced by Zika virus infection through the IRE1-XBP1 pathway. Front Cell Infect Microbiol. (2019) 9:480. 10.3389/fcimb.2019.00480 32039055 PMC6990111

